# Specialized pro-resolving mediators: key regulators in placental function and pregnancy complications

**DOI:** 10.1007/s00109-025-02561-w

**Published:** 2025-06-07

**Authors:** Luisa G. Sousa, Georgina Correia-da-Silva, Natércia Teixeira, Bruno Miguel Fonseca

**Affiliations:** 1 UCIBIO – Applied Molecular Biosciences Unit, Laboratório de Bioquímica, Faculdade de Farmácia, Universidade Do Porto, 4050-313 Porto, Portugal; 2 Associate Laboratory i4HB, Faculdade de Farmácia, Institute for Health and Bioeconomy, Universidade Do Porto, 4050-313 Porto, Portugal; 3 Laboratório de Bioquímica, Faculdade de Farmácia, Universidade Do Porto, Rua de Jorge Viterbo Ferreira, 4050-313 Porto, Portugal

**Keywords:** Specialized pro-resolving mediators (SPMs), Inflammation, Pregnancy complications, Lipoxin A4 (LXA4), Pregnancy supplementation

## Abstract

**Abstract:**

Specialized pro-resolving mediators (SPMs) are bioactive lipids derived from essential fatty acids that play a key role in resolving inflammation and modulating immune responses, thereby maintaining tissue homeostasis in various physiological contexts, including pregnancy. In healthy pregnancies, inflammation is a biological response necessary for vascular remodeling, embryo implantation as well as delivery and an increase in SPMs such as lipoxin A4 (LXA4) and resolvin D1 (RvD1) supports homeostasis and facilitates inflammation resolution. However, pregnancy complications such as spontaneous abortion, fetal growth restriction (FGR), and preeclampsia are often associated with disrupted SPM levels and receptor activity. In spontaneous abortion, altered SPM levels are linked to impaired endometrial receptivity, defective trophoblast invasion, poor epithelial-to-mesenchymal transition, and enhanced inflammation. Similarly, FGR is associated with reduced LXA4 levels, which contribute to placental vascular dysfunction and impaired trophoblast migration. Preeclampsia is characterized by dysregulated SPM levels and a pro-inflammatory environment, indicating insufficient resolutive activity. Therapeutic approaches to enhance SPM levels, such as aspirin-triggered lipoxins and omega-3 fatty acid supplementation, have demonstrated potential benefits. However, inconsistent clinical outcomes highlight the need for personalized treatment strategies. This review explores the role of SPMs in pregnancy, focusing on their molecular mechanisms and the development of targeted supplementation strategies to optimize their protective effects in managing high-risk pregnancies.

**Key messages:**

- Physiological pregnancies involve a gradual increase in SPM levels.
- LXA4 and RvD1 may have a context-dependent role in placentation by negatively regulating endometrial decidualization, trophoblast EMT and invasion, which contributes to spontaneous abortion, while positively regulating endothelial function, trophoblast survival and M2-macrophage polarization, which supports pregnancy.
- SPMs are essential to preserve endothelial integrity and support trophoblast proliferation, and appear downregulated in FGR.
- Preeclampsia is correlated with dysregulated SPM levels and a reduced LXA4/TNFα ratio, which suggests insufficient anti-inflammatory action.
- Therapeutic strategies that enhance SPMs production such as aspirin and DHA supplementation show considerable promise, particularly in preventing complications in high-risk pregnancies.

## Introduction

Decidualization of the human endometrium is a pivotal cyclic process, during which endometrial stromal cells (ESCs) undergo extensive remodeling and differentiate into specialized decidual cells under the influence of ovarian steroid hormones [1]. Besides the phenotypical transition from elongated fibroblast-like mesenchymal cells to round epithelial-like cells — known as epithelial-mesenchymal transition (EMT) — decidualized cells acquire unique cellular and biochemical properties that allow the expression and secretion of pregnancy-associated factors such as metalloproteinases, insulin-like growth factor-binding protein-1 (IGFBP1) and prolactin (PRL). This transformation prepares the endometrium by creating a nutrient-rich, immune-tolerant environment to support embryo implantation. On the other hand, placental trophoblast invasion into the decidua is critical for successful implantation and pregnancy [2]. Concurrently, EMT enhances trophoblast motility and invasiveness, enabling them to navigate the decidualized endometrium and fostering maternal–fetal communication [3]. These processes are associated with a highly pro-inflammatory environment and dynamic immune regulation. Along gestation, macrophages exert fundamental regulatory roles and placental macrophage types vary at different stages. M1-type macrophages, typically pro-inflammatory cells, are associated with the production of pro-inflammatory-related cytokines such as interleukin (IL)−6, IL-1β, and tumor necrosis factor-α (TNF-α), while M2-type macrophages are involved in promoting Th2 responses and related with anti-inflammatory responses [4, 5]. As pregnancy progresses, a shift towards an anti-inflammatory state ensures immune tolerance and protects the developing fetus. However, disruptions in the mechanisms controlling inflammation can result in excessive inflammatory responses, contributing to complications such as preeclampsia (PE) [6] and preterm labor [7], though the underlying processes are not yet fully understood [3].

In recent years, specialized pro-resolving mediators (SPMs) have emerged as critical regulators of inflammation in various physiological processes. SPMs actively suppress inflammation and promote tissue clearance, emphasizing that inflammation resolution is an active, dynamic process rather than a passive occurrence [8].Therefore, SPMs play a vital role in resolving inflammation, balancing immune activation, and actively promoting tissue repair. These mediators have been implicated in the regulation of inflammatory pathways associated with chronic diseases, including cardiovascular, metabolic, and autoimmune conditions [9]. They play diverse roles in infection control and wound healing [10]. Pregnancy, as a unique physiological state requiring maternal immune tolerance of the fetus alongside robust inflammatory responses to prevent infection, has garnered increasing interest in the role of SPMs [11].

SPMs are derived from essential omega-3 and omega-6 polyunsaturated fatty acids and include lipoxins (LX), resolvins (Rv), protectins (PD), maresins (MaR), and aspirin-triggered lipoxins (AT-LXs) [12]. Among SPMs, lipoxins (LXA4 and LXB4) play a crucial role in the class-switching mechanism, transitioning from pro-inflammatory leukotrienes to anti-inflammatory lipoxins, regulated through the modulation of lipoxygenase (LOX) enzymes, particularly 5-LOX [13]. Lipoxin A4 (LXA4) is an arachidonic acid metabolite, and in cooperation with its isomer lipoxin B4 (LXB4), play an active role in the regulation of inflammation. Biosynthesis involves the enzymes lipoxygenases—5-LOX, 12-LOX and 15-LOX. Lipoxin synthesis may be also triggered by aspirin or salicylic acid, which acetylates cyclooxygenase-2 (COX-2) [14]. Lipoxins exert their anti-inflammatory effects primarily via ALX/FPR2 receptor, by downregulating the expression of leukocyte-endothelial adhesion molecules and reducing tissue extravasion. SPMs also facilitate tissue repair by promoting non-phlogistic monocyte recruitment, apoptosis, and macrophage polarization towards the M2 phenotype, ensuring efficient clearance of apoptotic cells and restoring tissue homeostasis. Similarly, D-series resolvins (RvD) and E-series resolvins (RvE) are derivatives of docosahexaenoic acid (DHA) and eicosapentaenoic acid (EPA) and attenuate the production of pro-inflammatory cytokines such as IL-6 and IL-8 through key signaling pathways, including MAPK, AP-1, and NF-κB, while promoting the release of anti-inflammatory cytokines like IL-10. Resolvin signaling is mediated via a myriad of receptors such as ALX/FPR2, GPR32, BLT1 and ERV1 [15].

**Fig. 1:**
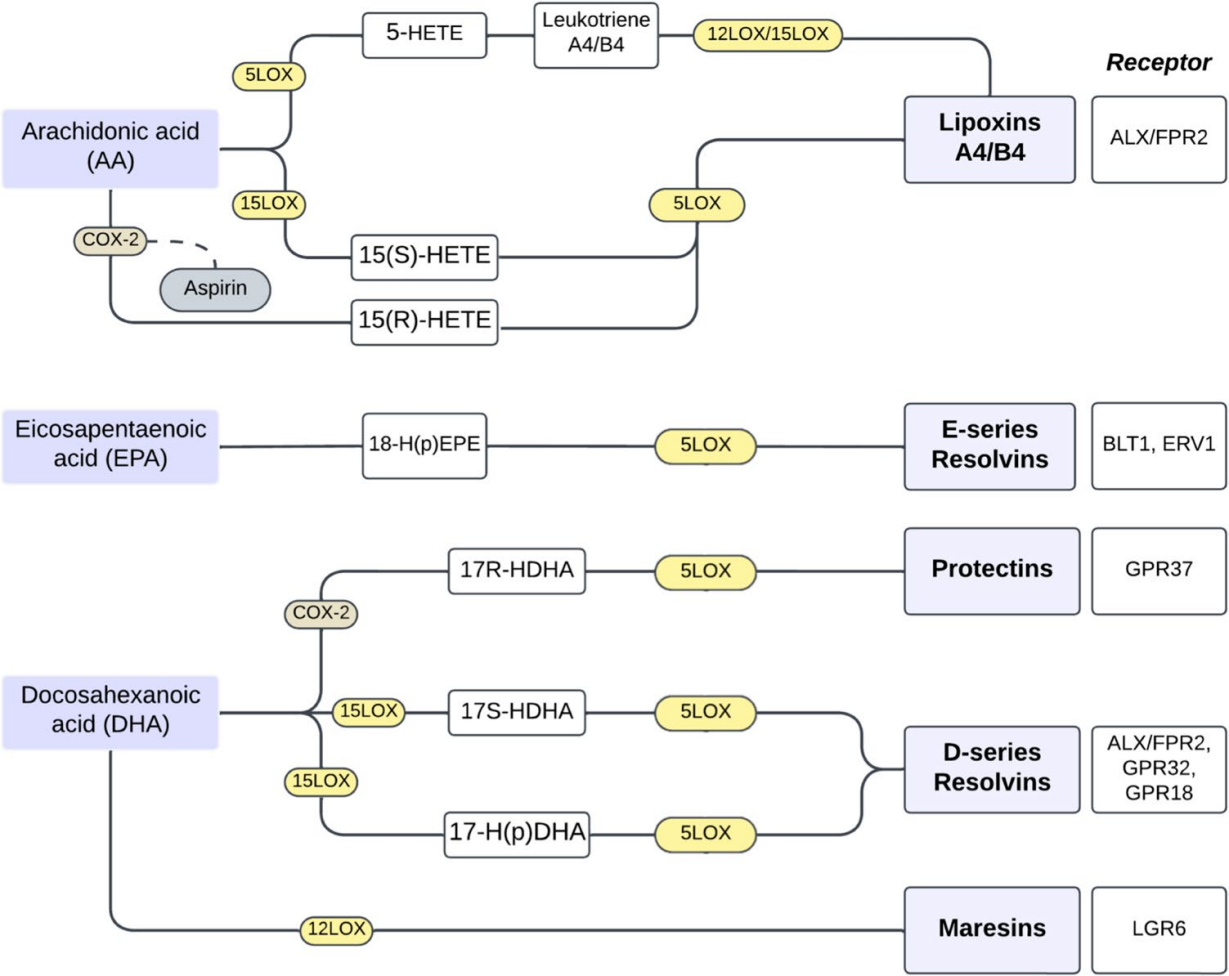
Biosynthetic pathways of SPMs leading to the production of lipoxins, resolvins, protectins, and maresins. SPMs are produced from their precursors arachidonic acid (AA), eicosapentaenoic acid (EPA), and docosahexaenoic acid (DHA). Key enzymes such as 5-LOX, 12-LOX and 15-LOX mediate the production of bioactive lipid mediators. Major known SPM receptors are shown on the right. Also depicted is the role of aspirin in inducing the specific aspirin-triggered lipoxin pathway via COX-2

LOX enzymes are critical in the biosynthesis of lipoxins and other SPMs, as they catalyze the oxygenation of the polyunsaturated fatty acids. The 5-LOX enzyme is encoded by the ALOX5 gene. It plays a key role in the conversion of arachidonic acid into leukotrienes and further into anti-inflammatory lipoxins under specific regulatory conditions, especially its cytoplasmic-nucleus location. The other LOX enzymes, 12- and 15-LOX are involved in the oxidation of polyunsaturated fatty acids to produce lipid intermediates that are precursors for lipoxins and other pro-resolving mediators. The SPM mediators are tighly regulated and the enzymes responsible for their biosynthesis play a pivotal role in maintaining the balance between pro-inflammatory and pro-resolving signals. This underscores the critical role of these enzymes and the SPM receptors in resolving inflammation and facilitating tissue repair. The major biosynthetic pathways of SPMs as well as their known receptors are described in Fig. 1.

The growing recognition of SPMs in resolving inflammation has led to an increasing number of studies exploring their role in pregnancy. Research demonstrates that SPMs contribute to maintaining pregnancy and can influence pregnancy-related conditions such as PE, gestational diabetes, and preterm birth [16]. However, there remains a significant gap in the knowledge regarding the roles of SPMs in normal versus complicated pregnancies. This review aims to address this gap by examining the physiological roles of SPMs during pregnancy and their effects on key processes, integrating findings from both clinical and non-clinical studies. By providing a detailed overview, this work will identify critical areas for future research and highlight potential therapeutic opportunities for improving maternal and fetal outcomes.

## Methods

### Eligibility criteria

Our inclusion criteria comprised original research articles published in English in peer-reviewed journals within the past 15 years (2010–2024). We excluded articles published before 2010, as well as conference abstracts, case studies, non-primary data articles (such as methods papers, opinion pieces, or proceedings), and reviews of any kind.

### Information sources and search strategy

This review involved a comprehensive search of the PubMed, SCOPUS, Web of Science, and Cochrane Library databases, conducted between September 20 and October 3, 2024. The search strategy combined keywords and controlled vocabulary terms (e.g., MeSH in PubMed and EMTREE in Embase) related to the main concepts of interest, namely, “pregnancy” and bioactive lipids such as “pro-resolving,” “lipoxin,” “resolvin,” “maresin,” and “protectin.” Only original research articles published in peer-reviewed journals were considered. The search was restricted to studies published in English between 2010 and 2024.

## Fluctuation of SPM levels in normal pregnancy

The reviewed literature consistently indicates that SPMs, particularly LXA4 and its receptor FPR2/ALX, generally show an increase during normal pregnancy, compared with non-pregnant women. Nevertheless, their expression patterns exhibit some variability [17–25]. FPR2 expression in the human endometrium peaks during the menstrual and late secretory phases, the latter corresponding to the post-implantation period, but significantly declines during the proliferative and early secretory phases. Additionally, LXA4 levels were found to progressively increase throughout pregnancy, without a marked rise either before or after labor [26]. In fact, myometrial LXA4 levels show no significant differences between laboring and non-laboring women, despite increased FPR2 expression in labor [26]. Another study found elevated LXA4 levels at the onset of labor and a negative correlation with neutrophil presence in the tissue, suggesting its role in modulating inflammation during childbirth [19]. Notably, machine learning approaches highlight the predictive potential of SPM measurements for preterm birth. RvD1 has emerged as a strong predictor of spontaneous preterm birth, whereas preterm births associated with placental abnormalities have been associated to elevated levels of pro-inflammatory leukotrienes. Among eicosanoids, LOX and CYP450 metabolites showed the greatest accuracy in identifying overall and spontaneous preterm birth [27]. These findings reveal inconsistencies when comparing laboring and non-laboring women, suggesting that SPM regulation is dynamic throughout pregnancy, though their exact roles in labor remain uncertain. A potential explanation for these differences lies in the variable biological stress of labor, which triggers adrenaline release. This adrenaline response may drive an increased demand for SPMs to mitigate excessive inflammation during labor.

FPR2/ALX expression is highest early in pregnancy and declines as gestation progresses [28]. RvD1 tends to show a modest increase from the second trimester to the third and during labor [29–31]; however, there are discrepancies between studies. Higher RvD1 levels are noted in term deliveries compared to preterm births in both humans and animal models [29]. Increased 12-LOX and 15-LOX, but not 5-LOX, was associated with the increase of LXA4 in active labor [19]. MaR1 shows a slight decline throughout pregnancy [32], whereas not enough information was found regarding other SPMs in order to draw significant conclusions.

**Fig. 2:**
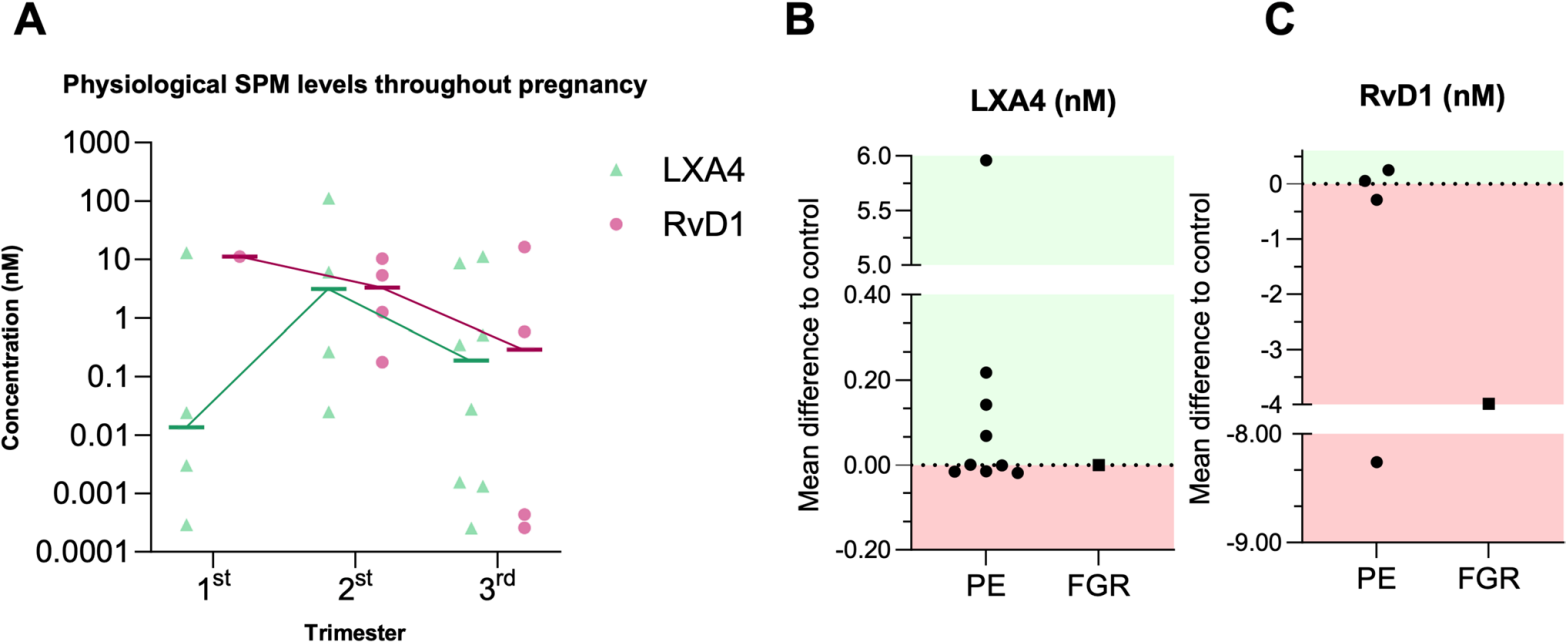
SPM levels during pregnancy and pathological conditions. **A** Physiological trend of LXA4 and RvD1 levels across the three pregnancy trimestres. Data points represent mean concentrations in nM for each study. Distinct trends are observed for both mediators. **B** Mean differences in LXA4 levels (nM) compared to controls in their respetive studies, for pregnancies complicated by preeclampsia (PE) and fetal growth restriction (FGR). **C** Mean differences in RvD1 levels (nM) compared to controls for PE and FGR. Positive and negative differences are highlighted in the green and red areas, respectively

A major challenge in the current literature is the lack of standardization across studies, leading to inconsistencies in results. Variations in measurement units, detection methods, and sample sources further complicate comparisons. Blood samples are often used because of their accessibility, but they may not accurately reflect local placental conditions. Despite these limitations, an illustrative graph built upon standardized data collected from blood serum levels of LXA4 and RvD1 (Fig. 2A), showing a marked elevation in LXA4 levels from the first to second trimester and a physiological decrease between the second and third trimesters for both LXA4 and RvD1. Nonetheless, all studies unequivocally show a need for LXA4 and RvD1 production as pregnancy progresses.

Pregnancy can be affected by complications such as PE, preterm labor, fetal growth restriction and spontaneous abortion. These conditions often involve disruptions in immune regulation, exacerbated inflammation, and impaired placental development. The involvement of dysregulated SPM levels in these pregnancy complications has been observed in numerous studies detailed in Table 1, which focuses exclusively on blood serum quantifications of LXA4 and RvD1, the most prominent SPMs studied in this field.

**Table 1:** Comparison of serum LXA4 and RvD1 Levels in complicated versus normal pregnancies, across trimestres

Pregnancy complication	Trimester at sampling	Serum LXA4	Serum RvD1	Study
**Preeclampsia**	1	164 vs 260 mg/L		**Vignes (2023)**
	1	3083 vs 8458 pg/ml		**Shanmugalingam (2020)**
	2	3083 vs 8743 pg/ml		
	3	146 vs 196 mg/L		**Vignes (2023)**
	3	201 vs 552 pg/ml		**Xu (2014)**
	3	2956 vs 9754 pg/ml		**Shanmugalingam (2020)**
	3	0.23 vs 0.18 ng/ml		**Gil-Villa (2012)**
	3	181 vs 91 ng/L		**Huang (2014)**
	3	290 vs 90 pg/ml		**Perucci (2016)**
	3	6 vs 3 ng/ml		**Dong (2016)**
	2	5 vs 4 log10/pg/ml	5 vs 4 log10/pg/ml	**Perucci (2021)**
	2		0.13 vs 0.12 pmol/100 μl	**Stephenson (2023)**
	3	5 vs 5 log10/pg/ml	5 vs 5 log10/pg/ml	**Perucci (2021)**
	3		3 vs 6 ng/ml	**Perucci (2020)**
**Fetal growth restriction**	1	68 vs 102 pg/ml		**Lipa (2017)**
	1		3 vs 4 µg/L	**Han (2024)**
	2		2 vs 3 µg/L	
	3		2 vs 4 µg/L	
**Chorioamnionitis**	3		30 vs 162 pg/ml	**Li (2020)**
**Metabolic disease**	2		0.0019 vs 0.002 ng/µl	**Maner-Smith (2022)**
**Preterm delivery**	3		57 vs 97 pg/ml	**Chen (2018)**
**Spontaenous abortion**	1	310 vs 1054 pg/ml		**Xu (2013)**

### Spontaneous abortion

Spontaneous abortion, also known as miscarriage, refers to the unintended loss of a pregnancy before the 20 th week and can occur due to a range of causes, including genetic abnormalities, hormonal imbalances, immune dysfunction, or issues related with the uterine environment. The mechanisms behind spontaneous abortion remain complex and not fully understood. Research suggests that factors such as dysregulated inflammation and improper implantation and placental development may contribute to its occurrence.

Recent studies have associated spontaneous abortion with elevated expression of FPR2 in villous cytotrophoblasts [33] and increased levels of LOX enzymes in endometrial samples from cases of spontaneous abortion [34]. Additionally, both LXA4 and RvD1 were found to significantly inhibit endometrial stromal decidualization in cultured human cells, via the downregulation of canonical decidualization markers IGFBP-1 and PRL [34]. These findings suggest that irregular inflammatory environments, which disrupt decidualization, may contribute to pregnancy failure. However, the role of LXA4 extends beyond this, as it has also been linked to trophoblast properties and placentation processes. In an in vitro study, LXA4 was found to upregulate epithelial markers while downregulating mesenchymal markers such as vimentin and fibronectin, thereby promoting increased trophoblast invasiveness [35].This suggests that LXA4 may inhibit EMT in first-trimester EVTs by reducing E-cadherin and β-catenin expression, potentially exerting anti-nidation effects.

Animal studies further confirm the involvement of SPMs, such as LXA4, in pregnancy. A study by Xu et al. demonstrated that administration of LXA4 during the peri-implantation period in mice led to anti-nidation effects, ultimately preventing successful implantation and pregnancy [35]. In contrast, administering LXA4 after implantation improved birth rates by reducing miscarriages [35]. Another study found that LXA4 administration led to complete inhibition of implantation [36]. In an LPS-induced miscarriage model, RvD1 significantly reduced the stillbirth rate from 64 to 32% [37]. Additionally, mouse studies have linked LXA4 to downregulation of caspase-1 expression and activity, a key inflammasome component, in the placenta, and the reduction in caspase-1 activity was also associated with decreased trophoblast apoptosis [38]. Similar results were observed with DHA, which inhibited caspase-1 and cathepsin-S, reducing preterm birth rates and suggesting a role for resolvins in these processes [29].

A compelling interaction between glucocorticoids and SPMs, particularly LXA4, in miscarriage has been observed. In mouse models, co-treatment with dexamethasone and LXA4 or its analogs effectively reversed many of the adverse effects of dexamethasone alone. This included restoration of maternal and fetal weights, increased placental capillary volume and blood space size, and reduced placental apoptosis, thereby alleviating growth restriction [39]. Remarkably, LXA4 administration also decreased lipid peroxidation, even in untreated control mice, underscoring its antioxidative potential. Dexamethasone appears to negatively regulate LXA4 production by activating 5-LOX, increasing leukotriene synthesis, and suppressing 15-LOX expression. These changes significantly inhibit LXA4 production, exacerbating inflammation. However, LXA4 supplementation counteracts these effects, reducing inflammatory markers and restoring homeostasis [13, 39]. LXA4 also plays a pivotal role in cortisol regulation by upregulating 11β-HSD2, an enzyme responsible for cortisol inactivation. This effect has been observed in trophoblast cells in both human and mouse models [40, 41]. By mitigating excessive cortisol levels, LXA4 may help protect against glucocorticoid-induced complications during pregnancy. However, evidence suggests a complex interplay where LXA4 and glucocorticoids may mutually downregulate each other, a dynamic that warrants further investigation to better understand and optimize therapeutic applications.

### Fetal growth restriction

Fetal growth restriction (FGR) is a critical healthcare challenge and a leading cause of late-pregnancy stillbirth, contributing significantly to neonatal mortality, prematurity, and long-term morbidity. In FGR cases, the placenta is often smaller and underdeveloped, showing marked morphological and functional abnormalities, including impaired trophoblast proliferation, increased apoptosis, and disrupted villous architecture with reduced branching of terminal villi. These placental deficiencies underscore improper placentation as a primary driver of FGR [42]. As such, of FGR as a strong predictor of perinatal death before 34 weeks [43].The impact of FGR extends beyond the neonatal period, as the offspring faces a heightened risk of cardiovascular, renal, and metabolic diseases, specifically hypertension and diabetes [44].

Reduced levels of LXA4 in the first trimester have been detected in women who later experienced either restricted or excessive fetal growth, suggesting a critical role for LXA4 in the regulation of fetal weight outcomes [45]. LXA4 was found to reverse the downregulation of cadherins, catenins, and cellular calcium levels in human umbilical vein endothelial cells (HUVECs), effectively preventing endothelial hyperpermeability. This effect was confirmed to be mediated through FPR2, as it was abolished by an FPR2 antagonist [46]. In trophoblast cell lines, silencing FPR2 significantly diminishes migratory and invasive potential. Downstream target genes such as STAT5B, SOCS3, and cadherins were identified as key mediators of FPR2’s role in trophoblast invasion and EMT [47]. However, conflicting results have emerged. For instance, one study reported that FPR2 suppression in trophoblast cells reduced apoptosis, activated the PI3 K/AKT pathway and impaired migration rates; however, it improved endothelial capillary-forming functions [33]. Another study found that FPR2 suppression increased endothelial permeability and reduced their tube formation [28]. In a first-trimester trophoblast model, LXA4 was shown to promote their migration [48]. This suggests that the effects of LXA4 and FPR2 signaling on trophoblasts and their interplay with endothelial cells may depend on yet unexplored context-specific factors (Fig. 2B).

Other SPMs are also implicated in FGR, specifically RvD1 (Fig. 2C). A recent study reported lower levels of RvD1 throughout pregnancy in women who later developed FGR. Mid-to-late pregnancy RvD1 levels demonstrated moderate predictive accuracy for FGR risk, particularly in older pregnant women, where advanced maternal age itself is a known risk factor [30]. In addition to SPM levels, research has highlighted a connection FPR2 signaling and negative pregnancy outcomes. For example, reduced FPR2 expression in the chorion has been observed in pregnancies involving small-for-gestational-age infants [47]. Conversely, upregulated FPR2 expression coupled with significantly lower levels of RvD1 has been associated with chorioamnionitis, a recognized cause of FGR [37]. FPR2 expression patterns are temporally regulated throughout pregnancy. In a mouse model, FPR2 mRNA levels are higher during the first trimester compared to term samples, with a marked decline as gestation progresses. Silencing FPR2 resulted in increased trophoblast proliferation, indicated by elevated choriogonadotropin expression. An apoptosis array revealed that FPR2 enhances the expression of apoptotic-related p53, caspase-8, and Bax, while anti-apoptotic Bcl2 levels remained unchanged. Additionally, silencing FPR2 in HUVECs reduced proliferation and impaired network formation, indicative of increased vascular permeability [28].

Taken together, the current evidence underscores the pivotal role of FPR2 in maintaining trophoblast and endothelial function in the placenta. Deregulated FPR2 expression and disruptions in SPM production, particularly LXA4, are likely to contribute to impaired placentation and FGR. As seen in spontaneous miscarriage, SPMs appear to have a dual and temporally regulated role in FGR. While conflicting findings exist regarding the beneficial or detrimental effects of increased SPMs, a physiological rise in SPMs post-implantation is essential to preserve endothelial integrity and support trophoblast proliferation. These complexities highlight the need for further investigation to clarify the precise mechanisms underlying FPR2 and SPM-mediated placental function.

### Preeclampsia

Preeclampsia (PE) is a pregnancy disorder characterized by new-onset hypertension occurring after 20 weeks of gestation. PE affects 2–7% of all pregnancies [17] and remains a leading cause of maternal and fetal morbidity and mortality [49]. PE is often associated with an increased risk of conditions such as diabetes mellitus and metabolic syndrome later in life. The condition is driven by key pathological processes, including endothelial dysfunction, placental insufficiency, inflammation, oxidative stress, and heightened immune responses. Severe cases of PE can result in organ damage, particularly renal and vascular dysfunction, where inflammatory and angiogenic factors contribute to glomerular endotheliosis, a life-threatening complication [50].

Altered inflammation of maternal–fetal interface is a critical factor in the pathophysiology of PE with markedly elevated levels of pro-inflammatory factors and a shift from M2 macrophage phenotype to M1 phenotype in PE placentas [51, 52]. Given the inflammatory basis of PE, the role of SPMs in this disorder is unsurprising. Elevated levels of LXA4 have been reported in PE patients compared to gestation-matched controls (Fig. 2B), suggesting a compensatory response to excessive inflammation [17, 20, 22, 32]. A reduced LXA4/TNFα and LXA4/IL-1β ratio in PE patients suggests insufficient anti-inflammatory effects, likely due to exacerbated, chronic inflammation in these women [17]. However, other studies have reported decreased LXA4 levels in both serum and placental tissues of PE patients, with reduced expression of LOX enzymes, indicating impaired SPM production in PE [40, 41].

Findings regarding resolvins in PE are also contradictory. A 2020 study reported lower RvD1 levels in PE, accompanied by a reduced resolvin/leukotriene ratio, indicating a pro-inflammatory shift [32]. In contrast, another study found elevated plasma levels of RvD1 during early gestation (12–19 weeks), which decreased at later stages (30–34 weeks) in women who developed PE, compared to those who remained normotensive [53]. Elevated early gestational RvD1 levels were also observed in women who later developed preterm PE [31] (Fig. 2C). Furthermore, higher resolvin levels have been documented in PE patients with metabolic syndrome, characterized by hypertension, hyperglycemia, dyslipidemia, and abdominal obesity [49]. Although maresins are less studied in PE, reduced median levels in PE patients suggest impaired inflammation resolution [54].

SPMs mitigate inflammation by modulating key signaling pathways, including NF-κB pathway. Blocking LXA4 signaling in a PE mouse model exacerbated the symptoms, increasing blood pressure, proteinuria, and FGR [41]. Moreover, FPR2 negatively correlates with NF-κB expression, reinforcing its role in dampening inflammation [20], as further supported by another study [55]. In addition, a strong correlation between RvD1 levels and blood pressure in PE has been observed, indicating a role for SPMs in vascular regulation [49]. The detection of eicosanoid levels in a study allowed researchers to distinguish between PE patients with term and preterm births, with increased RvD1 levels seen in patients later diagnosed with preterm PE [31]. Lipid peroxidation markers, such as thiobarbituric acid reactive substance and 8-isoprostane, were significantly elevated in PE plasma, exacerbating endothelial inflammation. Treatment with LXA4 in PE plasma-conditioned HUVECs reduced PMN adhesion by up to 91%, demonstrating potent anti-inflammatory effects [18]. Currently, the only definitive treatment for PE is induced delivery, highlighting the urgent need for alternative therapies. The anti-inflammatory and vascular regulatory properties of SPMs position them as promising candidates for therapeutic exploration, warranting further investigation in clinical trials.

**Fig. 3:**
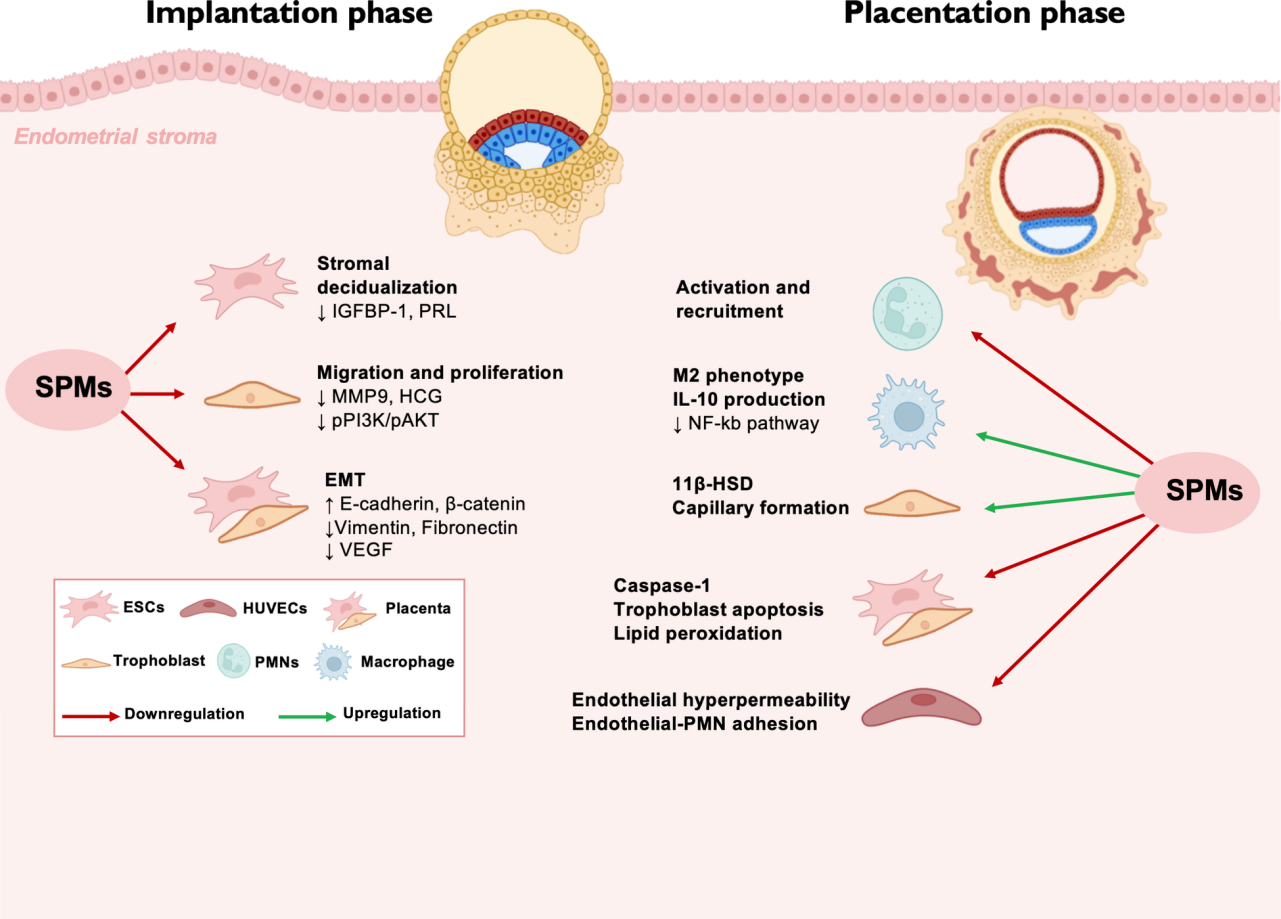
Specialized Pro-resolving Mediators (SPMs) in the implantation and placentation phases of pregnancy. In the implantation phase, SPMs downregulate stromal decidualization markers (IGFBP-1, PRL), migration and proliferation factors (MMP9, HCG, pPI3 K/pAKT), and epithelial-mesenchymal transition (EMT), while increasing markers such as E-cadherin, β-catenin, vimentin, fibronectin, and VEGF. During placentation, SPMs promote macrophage activation and recruitment, support the M2 phenotype by increasing IL-10 production, and inhibit the NF-κB pathway. Furthermore, SPMs enhance capillary formation and mitigate adverse processes such as caspase-1 activation, trophoblast apoptosis, lipid peroxidation, endothelial hyperpermeability, and endothelial-polymorphonuclear cell (PMN) adhesion. Red arrows indicate downregulation while green arrows indicate upregulation. The figure label represents the cell types involved, including endometrial stromal cells (ESCs), human umbilical vein endothelial cells (HUVECs), trophoblasts, macrophages, and PMNs

Overall, SPMs are potentially involved in several processes during early and late pregnancy, modulating stromal decidualization, trophoblast migration and proliferation, immune modulation as well as their contribution to placental development and vascular remodeling during the placentation phase. A dysregulation in SPM production can contribute to poor placentation, growth restriction or even spontaneous abortion (Fig. 3).

## SPM-increasing supplementation and clinical perspectives

Recently, clinical trials have focused on aspirin-mediated lipoxin increase or omega-3 supplementation to enhance placental resolvin production. These interventions are of interest due to their potential roles in modulating SPM and related pathways to maintain maternal–fetal homeostasis and controlling inflammation. Table 2 shows the main details of all the clinical trials analyzed, including study design and dosages, population characteristics and their main outcomes.

**Table 2:** Clinical trials evaluating the effects of aspirin and omega-3 supplementation on the production of lipid mediators in pregnancy and their outcomes

Author, Year	Key findings	Sample Characterization	Participants	Source tissue	Measured lipid mediator
**Pietrantoni E (2014)**	Supplementation increased levels of ω−3 FAs and DHA in the blood and counteracted the physiological decline in untreated group. Statistically reduced incidence of membrane rupture and a longer duration of pregnancy	C: 200 mg DHA capsule daily from the 8 th week of pregnancy until delivery (n = 129) S: Placebo (olive oil, n = 126)	Age: DHA group: 30.86 ± 4.18 Placebo group: 29.92 ± 4.80 Gestational week at sampling: 17, 25 and 38 weeks	-	-
**Keelan JA (2015)**	Supplementation increased placental DHA levels by 80%. RvD1, 17R-RvD1, RvD2 and PD1 were not significantly increased. Serum DHA levels were correlated with placental DHA but not with placental RvD1 or RvD2	C: Supplemented daily from the 20 th week with fish oil (n = 22) corresponding to 3,7 g of ω−3 PUFAs S: placebo (olive oil, n = 28)	Age: Fish oil group: 29.8 ± 4.0 Placebo group: 32.5 ± 3.7 Gestational week at sampling: 36 weeks and birth	Placenta	RvD1 C:29,66 ± 8,56 pg/mg S:33,94 ± 8,97 pg/mg RvD2 C:50,66 ± 5,91 pg/mg S: 54,73 ± 9,18 pg/mg PD1 C:29,66 ± 8,76 pg/mg S:30,27 ± 11,42 pg/mg
**Gonzalez-Brown (2020)**	Obese (BMI > 30) women have lower levels of 15-epi-LXA4 compared to non-obese women. Daily aspirin increases 15-epi-LXA4 in obese women compared to non-obese women at high risk for developing PE	C: Second trimester, high risk PE (n = 42), S: high risk PE BMI > 30 (n = 21) Daily low dose aspirin (60 mg)	Age: unknown Gestational week at sampling: 24–28	Blood/serum	LXA4 percent change after daily aspirin C: 3,31% S:11,7%
**Shanmugalingam R (2020)**	Plasma LXA4 is lower in high-risk PE. Aspirin treatment increases AT-LXA4, IL-10 and reduces pro-inflammatory cytokines. Aspirin 150 mg was more effective	C: Low-risk PE S1: High-risk PE S2: High-risk PE + aspirin Aspirin was administered at a dose of 100 or 150 mg	Age: C: 32 ± 5.3 S: 33 ± 5.5 Gestational week at sampling: 12,16,20,24,28,32 and 36	-	-
**Gonzalez-Brown VM (2021)**	Patients taking daily aspirin had significantly increased levels of AT-LXA4 compared to placebo. Patients who developed PE had decrease in AT-LXA4 compared to those without PE	C: High-risk PA, placebo (n = 29) S: High-risk PA + aspirin (n = 63) Aspirin was administered at a dose of 60 mg	Age: C: 23.5–30 S: 23–30 Gestational week at sampling: 13–26, 24–28, 34–38	Blood/serum	LXA4 C: 77,75 (13–26), 50,66 (24–28) and 56,6 (34–38) pg/ml S: 188,67 (13–26), 1698,11 (24–28), 2264,15 (34–38) pg/ml
**Otlu Ö (2023)**	An elevation of one unit (ng/mL) of LXA4 was associated with a 0.926-fold decrease in likelihood of obesity. Obese women in second trimester of pregnancy had decreased levels of LXA4	C: Second trimester, control (n = 30) S: Second trimester, BMI > 30 (n = 30)	Age: C:20–43 S: 21–43 Gestational week at sampling:15–28	Blood/serum	LXA4 C: 39 ng/ml S: 30 ng/ml
**Stephenson DJ (2023)**	Eicosanoid levels were able to stratify between PE patients with term and preterm births. Patients later diagnosed with PE with a preterm showed increased levels of RvD1	C: Second trimester, control (n = 31) S: Second trimester, PE (n = 26)	Age: C:28.2 ± 5.2 S:30.3 ± 7.6 Gestational week at sampling: C:14.89 ± 3.8 S:16.5 ± 5.2 84.6% of S and 45.1% of C were using prescribed aspirin	Blood/serum	RvD1 C:0,126 pmol/100 μl S: 0,131 pmol/100 μl
**Vignes K (2023)**	LXA4 significantly lower in high-risk participants at enrollment. LXA4 decreased in both groups, but the decline was significantly less in the aspirin group	C: Low risk PE (n = 56) S1: High-risk PE + 81 mg aspirin (n = 47) S2: High-risk PE + 162 mg aspirin (n = 57)	Age: C: 30.04 S1: 30.32 S2: 29.19 Gestational week at sampling: 11–16,28–32	Blood/serum	LXA4 C: 260,31 (11–16), 196,24 (28–32) mg/L S1:163,62 (11–16), 146,13 (28–32) mg/L S2:173,75 (11–16), 168,63 mg/L
**Han Y (2024)**	Lower RvD1 levels throughout pregnancy in women who developed FGR. Age was linked with elevated risk of FGR. RvD1 had a modest capability to estimate FGR risk in elderly pregnant women	C: Pregnant women without FGR (n = 140) S: Pregnant women with FGR (n = 25)	Age: C: 37.0 S: 39.0 Gestational week at sampling: 10–13, 20–23, 30–33	Blood/serum	RvD1 C: 4,73 (10–13), 3,90 (20–23), 4,47 (30–33) µg/L S: 3,45 (10–13), 2,40 (20–23), 2,74 (30–33) µg/L

### Aspirin-triggered lipoxins

Aspirin is currently used to prevent pregnancy complications, particularly in high-risk populations prone to hypertensive disorders. The mechanism for increasing lipoxin levels involves inhibition of COX-1, the enzyme responsible for converting arachidonic acid to prostaglandins, while promoting acetylated COX-2-mediated production of 15-epi-lipoxins, AT-LXs. These are structurally modified versions of lipoxins, where the configuration of the hydroxyl group at position 15 is 15(R) rather than 15(S). This modification confers a significanlty higher resistance to degradation and therefore a longer half-life. Additionally, this modification alters its enzymatic activity, to generate 15(R)-HETE instead of prostaglandins. AT-LXA4 is the most extensively studied variant [18]. Interestingly, direct administration of AT-LXA4 has shown greater efficacy than aspirin or salicylic acid. For instance, pretreatment with AT-LXA4 enhanced monocyte adhesion to HUVECs and significantly reduced PMNs adhesion, outperforming aspirin or salicylic acid pretreatment [55]. Additionally, in a mouse model, AT-RvD1 treatment decreased preterm birth rates caused by inflammation, likely through reduced uterine and cervical expression of pro-inflammatory cytokines such as IL-1β and TNF-α [56].

Clinical studies support these findings. Aspirin doses between 60 and 162 mg daily have demonstrated positive effects on SPMs biosynthesis in high-risk pregnancies. For instance, 60 mg of aspirin increased AT-LXA4 biosynthesis in obese women at heightened risk of PE [57]. A study on PE pathology found that plasma LXA4 levels were lower in high-risk PE patients. Similarly, a placebo-controlled trial demonstrated that daily aspirin (60 mg) significantly elevated AT-LXA4 levels compared to baseline and placebo groups. Among patients who developed PE, AT-LXA4 levels were reduced compared to those who remained PE-free, indicating aspirin’s role in enhancing SPM-mediated resolution pathways and preventing complications in high-risk groups [58]. A recent 2023 study in low- and high-risk patients observed that while lipoxin concentrations declined during pregnancy, aspirin supplementation (81 or 162 mg daily) significantly mitigated this decline compared to unexposed groups [24]. Aspirin at 150 mg was more effective than lower doses in increasing AT-LXA4 and IL-10 levels while reducing pro-inflammatory cytokines in these patients [23]. These findings highlight aspirin’s potential to modulate SPM levels and improve outcomes in pregnancies at risk of inflammatory complications.

### Omega-3 supplementation

Omega-3 supplementation has been shown to elevate placental levels of DHA and SPM precursors, but this does not consistently translate into higher levels of active SPMs or a reduced inflammatory profile, raising questions about its overall effectiveness [59]. For instance, one study found a higher pro-inflammatory profile in the placenta despite omega-3 supplementation. However, it also reported increased levels of the SPMs PD1, RvD1, RvD2, and LOX enzymes [15].

In contrast, DHA supplementation during the second and third trimesters has been shown to counteract physiological declines in maternal DHA levels. Notably, supplementation was associated with a reduced incidence of premature membrane rupture and extended pregnancy duration [60]. Another trial reported an 80% increase in placental DHA levels and two- to threefold increases in SPM precursors such as 18-HEPE and 17-HDHA, although active SPMs like RvD1, RvD2, and PD1 remained unchanged. Maternal DHA levels correlated strongly with placental DHA and 17-HDHA but not with active SPMs. Interestingly, maternal plasma levels of RvD1 and RvD2 were significantly associated with cord plasma levels of RvD2 and better outcomes in high-risk maternal–fetal health conditions, suggesting that omega-3 supplementation may enhance SPM activity in at-risk scenarios [61]. These findings highlight that although omega-3-rich diets or supplements can increase SPM precursor levels, their anti-inflammatory benefits may depend on personalized timing and dosing according to pregnancy risk factors. Additional clinical trials are needed to clarify the optimal use of omega-3 supplements to improve pregnancy outcomes.

## Conclusion

This review highlights the dynamic and multifaceted roles of SPMs in both normal pregnancies and various pregnancy complications. SPMs, including LXA4 and RvD1, typically increase during normal pregnancy, supporting immune homeostasis and regulating inflammation. However, the precise patterns of SPM levels fluctuate, particularly during labor, highlighting the complexity of their regulation in different pregnancy stages. In contrast, pregnancy complications often show altered SPM levels, with inconsistent findings across studies. The timing of SPMs changes appears to be a critical factor, particularly in conditions like spontaneous abortion, where the onset of inflammation and its resolution may be disrupted. These discrepancies also suggest potential interactions between SPMs and other compounds, such as glucocorticoids, which may influence SPM function and overall pregnancy outcomes. For example, studies with SPM and dexamethasone co-treatment have demonstrated that SPMs can help mitigate inflammation-associated risks, thus protecting maternal and fetal health.

Therapeutic strategies that enhance SPMs synthesis, such as aspirin-triggered lipoxins and omega-3 supplementation, show considerable promise, particularly in preventing complications in high-risk pregnancies. Aspirin has been shown to effectively increase anti-inflammatory AT-LXA4 levels in high-risk preeclampsia patients, while omega-3 supplementation is associated with higher levels of SPM precursors in both maternal and fetal circulations. Despite these promising findings, inconsistencies across studies call for the development of standardized SPMs detection methods and emphasize the need for personalized treatment regimens, where timing and dosage are tailored to the specific risks of each pregnancy.

Future research should focus on further elucidating the molecular pathways by which SPMs regulate pregnancy-related processes and how they interact with other inflammatory mediators in pregnancy complications. Additionally, clinical trials using a targeted approach with optimized SPMs supplementation strategies based on individual pregnancy risks could significantly improve outcomes in high-risk pregnancies. Advancing our understanding of SPMs and their therapeutic potential will provide new avenues for the management and prevention of pregnancy complications, ultimately improving maternal and fetal health.

## Data Availability

The data generated during the current study are available from the corresponding author upon reasonable request.

## Abbreviations

EMT: Epithelial-mesenchymal transition
SPM: Specialized pro-resolving mediator
LX: Lipoxin
Rv: Resolving
MaR: Maresin
PD: Protectin
AT-LX: Aspirin-triggered lipoxin
LOX: Lipoxygenase
COX: Cyclooxygenase
PMN: Polymorphonuclear cell
ALX/FPR2: Ipoxin receptor
IL: Interleukin
GPR: G-protein coupled receptor
RSA: Recurrent spontaneous abortion
EVT: Extravillous trophoblast
DHA: Docosahexaenoic acid
FGR: Fetal growth restriction
HUVEC: Human umbilical vein endothelial cell
HCG: Human chorionic gonadotropin
PE: Preeclampsia
